# Implications of Identifying Additional Cerebral Metastases during Gamma Knife Radiosurgery

**DOI:** 10.1155/2012/748284

**Published:** 2011-08-15

**Authors:** Toral R. Patel, Ali K. Ozturk, Jonathan P. S. Knisely, Veronica L. Chiang

**Affiliations:** ^1^Department of Neurosurgery, Yale University School of Medicine, P.O. Box 208082, New Haven, CT 06520, USA; ^2^Department of Therapeutic Radiology, Yale University School of Medicine, P.O. Box 208040, New Haven, CT 06520, USA; ^3^Yale Cancer Center, Yale University School of Medicine, P.O. Box 208028, New Haven, CT 06520, USA

## Abstract

*Introduction*. Gamma Knife radiosurgery (GK-SRS) is commonly used to treat cerebral metastases. Although additional intracranial metastases are often found on the day of GK-SRS, the significance of finding them is unknown. *Methods*. A retrospective review of 133 patients undergoing GK-SRS for cerebral metastases was performed. The change in number of metastases detected between initial referral magnetic resonance imaging (MRI) and subsequent treatment MRI was quantified. Multivariate and Kaplan-Meier analyses were employed to examine the significance of identifying additional lesions. *Results*. Additional lesions were identified in 41% of patients. An increasing number of metastases on referral MRI (*P* = 0.001) and the presence of progressive systemic disease (*P* = 0.003) were predictive of identifying additional metastases. Median survival was 6.9 months for patients with additional metastases, compared to 12.1 months for patients without additional metastases (hazard ratio 1.56, *P* = 0.021). *Conclusions*. Identifying additional metastases on the day of GK-SRS may add important prognostic information.

## 1. Introduction

Approximately 15–40% of cancer patients will develop metastatic lesions to the brain [1, 2]. Indeed, metastatic intracranial disease is 10-fold more common than primary brain tumors [3]. The presence of metastatic intracranial disease is still uniformly considered to be a poor prognostic indicator [4]. In the oncology literature, the treatment of cerebral metastases with whole-brain radiotherapy (WBRT) and/or Gamma Knife radiosurgery (GK-SRS) has not significantly changed overall survival [4]. However, more recent radiosurgical studies suggest that some patients with intracranial metastases are surviving longer (≥4 years) [5, 6]. Nonetheless, very few factors have been reproducibly identified as predictive of outcome. Strategies to further improve survival may rest on the accurate identification of novel prognostic factors. 

Additional intracranial metastases are often identified on the day of GK-SRS [7]. However, little is known about the prognostic significance of identifying additional cerebral metastases during GK-SRS. To understand this relationship, we retrospectively analyzed data from a cohort of patients who underwent GK-SRS for cerebral metastases, to determine which factors were predictive of identifying additional cerebral metastases and whether the identification of additional cerebral metastases had an effect on survival.

## 2. Materials and Methods

### 2.1. Study Population

We performed an Institutional-Review-Board-approved retrospective review of medical records at Yale-New Haven Hospital and the Yale-New Haven Gamma Knife Center for all patients who underwent their first GK-SRS treatment for intracranial metastases between May 1, 2002 and March 30, 2006. All patients gave their informed consent prior to inclusion in the study.

All patients were referred accompanied by diagnostic 1.5 Tesla gadolinium-enhanced axial magnetic resonance images (MRI); imaging parameters were otherwise heterogeneous. The number of pre-GK-SRS metastases was determined based on these referral scans. On the day of GK-SRS, all patients underwent gadolinium-enhanced axial MRI on a 1.5 Tesla magnet with a 3-dimensional spoiled gradient recalled acquisition sequence using 2 mm cuts with no gap. All GK-SRS procedures were performed on a model C Leksell Gamma Knife (Elekta Instruments). All identified metastases were treated. The GK-SRS dose delivered to the tumor margin ranged from 18 to 24 Gy, prescribed to the 40–70% isodose line. All patients who underwent GK-SRS had a Karnofsky Performance Score ≥70.

We collected data on demographics (age, sex), primary disease (pathology, systemic control at the time of GK-SRS), chemotherapy, cranial surgery, time between diagnostic and treatment scans, WBRT, and number of metastases (pre-GK-SRS and GK-SRS). Systemic control was defined as no detectable progression of primary tumor in organ systems outside of the central nervous system, at the time of GK-SRS. We accessed the Connecticut Tumor Registry and Social Security Administration Death Master File to obtain dates of all patient deaths that occurred by July 1, 2008.

### 2.2. Data Analysis

Statistical analyses were performed using STATA programming software (version 9.0, StataCorp LP, College Station, TX, USA). The primary endpoint was the change in number of metastases detected between the referral diagnostic pre-GK-SRS MRI and the subsequent treatment MRI (“Delta Mets”). The predictor variables that were examined included age, sex, primary pathology, systemic control, chemotherapy, cranial surgery, time between diagnostic and treatment scans, WBRT, and number of metastases (both pre-GK-SRS and at GK-SRS). 

Means, standard deviations, and medians were calculated for categorical variables. The 10th, 25th, 50th, 75th, and 90th percentiles were calculated for continuous variables. For subgroup analyses, we used the Fisher exact test for categorical variables and the Wilcoxon signed-rank test for continuous variables. The Kaplan-Meier method was used to examine the effect of a change in the number of metastases on overall survival. For all analyses, probability values <0.05 were considered significant, and probability values ranging from 0.10 to 0.05 were considered trends. 

## 3. Results

### 3.1. Study Population

One hundred and thirty-three patients with intracranial metastases were treated with GK-SRS during the study period. Median patient age was 58.7 years (range 29.0–85.5 years). The majority of patients were female (65%). The most common primary pathology was lung cancer (47%), followed by breast cancer (20%), melanoma (11%), and renal cancer (9%). The median time between pre-GK-SRS and treatment scans was 31 days (range 4–81 days). The median number of metastases identified on pre-GK-SRS MRI was 1 (range 1–10); the median number of metastases identified on treatment MRI was 2 (range 1–21). Fifty-two percent of patients had progressive systemic disease at the time of GK-SRS. Forty-seven percent of patients had WBRT prior to GK-SRS, 12% of patients had WBRT after GK-SRS, and 41% of patients did not undergo WBRT at any time. Twenty-six percent of patients had cranial surgery and 98% of patients were undergoing chemotherapy (Table 1). Median follow-up duration was 10.4 months (range 0.3–72.4 months).

### 3.2. Univariate Analysis

Fifty-four of the 133 patients (41%) had additional metastases identified on their treatment scan, as compared to their initial diagnostic scan. The median number of additional metastases identified within this subgroup was 2 (range 1–11). On univariate analysis, the number of pre-GK-SRS metastases was predictive of additional metastases being identified (*P* < 0.001). Furthermore, the presence of progressive systemic disease was also predictive of identifying additional metastases. Specifically, 65% of patients who had additional metastases identified on their treatment scan had progressive systemic disease at the time of GK-SRS (*P* = 0.021). Age, sex, primary pathology, WBRT, cranial surgery, chemotherapy, and time between scans did not have a statistically significant effect on the identification of additional metastases (Table 1).

### 3.3. Multivariate Analysis

**Figure 1:**
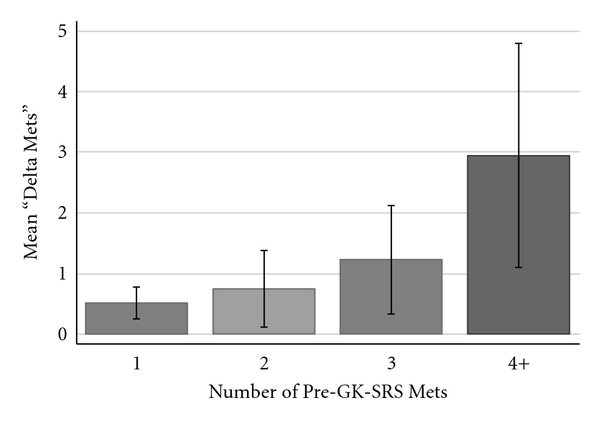
Bar graph demonstrating the relationship between the number of pre-GK-SRS metastases identified and the number of additional metastases (“Delta Mets”) identified at the time of GK-SRS. *Error bars* are shown.

**Figure 2:**
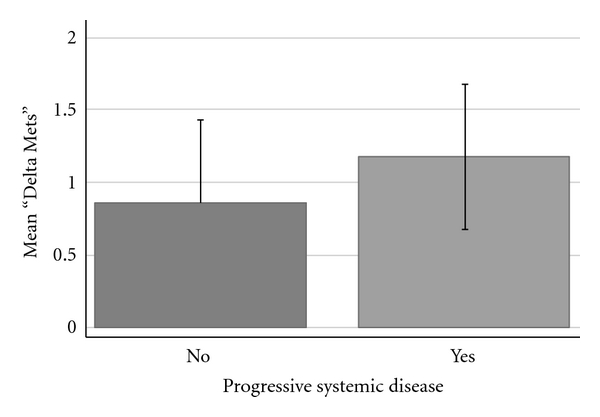
Bar graph demonstrating the relationship between progressive systemic disease and the number of additional metastases (“Delta Mets”) identified at the time of GK-SRS. *Error bars* are shown.

Based on the results of the univariate analysis, a stepwise multivariate regression analysis was performed. All predictor variables with *P* < 0.150 were analyzed and those variables with *P* < 0.100 were included in the final multivariate model. The multivariate analysis confirmed that both the number of initially identified (pre-GK-SRS) lesions (*P* = 0.001; Figure 1) and the presence of progressive systemic disease (*P* = 0.003; Figure 2) were independently predictive of identifying additional cerebral metastases. The multivariate analysis also confirmed that the identification of additional metastases was independent of all other predictor variables analyzed.

### 3.4. Survival Analysis

**Figure 3:**
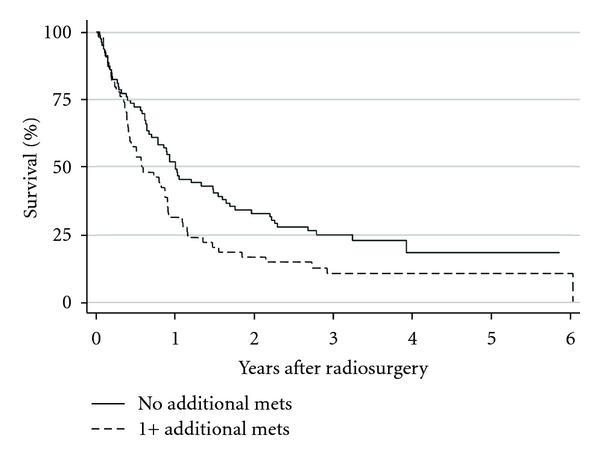
Kaplan-Meier plot of overall survival after GK-SRS for all 133 patients, stratified by the number of additional metastases identified at the time of GK-SRS.

**Figure 4:**
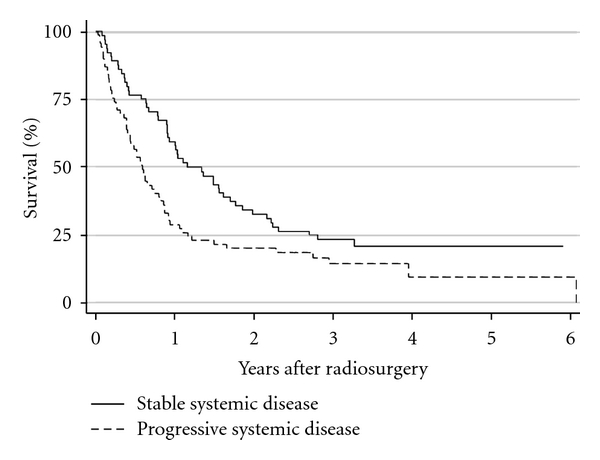
Kaplan-Meier plot of overall survival after GK-SRS for all 133 patients, stratified by the presence of progressive systemic disease at the time of GK-SRS.

To examine the effect of identifying additional cerebral metastases on survival, the Kaplan-Meier method was employed. For patients in whom no additional metastases were identified, the median survival was 12.1 months; however, for patients in whom ≥1 additional metastases were identified, the median survival was only 6.9 months (*P* = 0.021, hazard ratio = 1.56; Figure 3). Cox multivariate regression analysis revealed that the significant decrease in survival for patients in whom additional metastases were identified is independent of the initial (pre-GK-SRS) number of metastases identified (*P* = 0.024). Of note, the Kaplan-Meier curves for survival based on presence of progressive systemic disease are essentially identical to those obtained from analysis of survival based on additional metastases (Figure 4). Specifically, for patients with stable systemic disease, median survival was 13.8 months, while for patients with progressive systemic disease, median survival was 7.0 months (*P* = 0.012, hazard ratio = 1.63).

## 4. Discussion

In our retrospective analysis of 133 patients undergoing GK-SRS for intracranial metastases, we found that 41% of patients had additional metastases identified on their treatment MRI as compared to their diagnostic MRI. Furthermore, we found that the identification of new metastases correlated with both the number of pre-GK-SRS metastases identified and the presence of progressive systemic disease. Kaplan-Meier analysis revealed a significant decrease in survival for those patients in whom additional metastases were identified. Moreover, Kaplan-Meier analysis also revealed that patients with progressive systemic disease had similar survival profiles to patients in whom additional cerebral metastases were identified.

To date, patients undergoing GK-SRS for intracranial metastases have largely been stratified into two groups, based on the number of metastases they harbor. Although the cutoff values for these groups have varied (2 versus 3 versus 4 metastases), the literature has been nearly uniform in stating that those patients who harbor more metastases have a significantly worsened prognosis [1, 3, 8–10]. Moreover, the number of intracranial metastases present is often used as a surrogate for the aggressiveness of a patient's systemic disease, with more intracranial metastases signifying more aggressive systemic disease [1, 3]. Our results suggest that the change (or lack thereof) in number of intracranial metastases may be an additional prognostic marker. This data also implies that for patients in whom additional intracranial lesions are identified (on the day of GK-SRS), restaging of disease or changing systemic therapies may be warranted.

The limitations of this study include the inherent selection biases present when analyzing a cohort of patients treated at a single academic medical center. However, our demographic and clinical patient profiles are similar to those previously reported and we have no reason to believe that our results are not applicable to all patients with metastatic intracranial disease. Additionally, precise imaging parameters were not available for the initial diagnostic scans, as they were largely performed at outside institutions. However, this heterogeneous assortment of referral, diagnostic imaging scans is likely encountered in the majority of large academic medical centers, and therefore the results of this study are likely applicable to institutions similar to ours.

## 5. Conclusions

This study highlights the dynamic nature of metastatic intracranial disease. Identification of additional cerebral metastases on the day of GK-SRS yields important prognostic information which may be useful in directing patient care. The ability to perform high-resolution MR imaging, unique to GK-SRS, facilitates the ability to obtain this prognostic factor.

##  Disclosure

The authors report no conflict of interests concerning the materials or methods used in this study or the findings specified in this paper.

## Figures and Tables

**Table 1:** Study population and treatment characteristics*.

Treatment	Study cohort, *n* = 133	No additional mets, *n* = 79	≥1 additional mets, *n* = 54	*P*
*Age, years*				
Median	58.7	57.5	58.8	0.783
Range	29.0–85.5	29.0–85.5	30.6–82.6	
*Sex*				
Male	47 (35)	24 (30)	23 (43)	0.196
Female	86 (65)	55 (70)	31 (57)	
*Primary pathology*				
Lung	62 (47)	37 (47)	25 (46)	1.000
Breast	27 (20)	16 (20)	11 (20)	1.000
Melanoma	15 (11)	6 (8)	9 (17)	0.161
Renal	12 (9)	9 (11)	3 (6)	0.359
Other	17 (13)	11 (14)	6 (11)	0.792
*WBRT*				
Pre-GK-SRS	63 (47)	34 (43)	29 (54)	0.289
Post-GK-SRS	16 (12)	9 (11)	7 (13)	0.792
Neither	54 (41)	36 (46)	18 (33)	0.208
*Craniotomy*				
Yes	34 (26)	23 (29)	11 (20)	0.314
No	98 (74)	56 (71)	43 (80)	
*Chemotherapy*				
Yes	130 (98)	78 (99)	52 (96)	0.566
No	3 (2)	1 (1)	2 (4)	
*Time between scans, days*	31 (4–81)	29 (4–81)	37 (7–78)	0.111
*Number of metastases*				
Pre-GK-SRS	1 (1–10)	1 (1–9)	2 (1–10)	**<0.001**
GK-SRS	2 (1–21)	1 (1–9)	4 (2–21)	**<0.001**
*Progressive systemic disease*				
Yes	69 (52)	34 (43)	35 (65)	**0.021**
No	64 (48)	45 (57)	19 (35)	

*Number of patients (%), unless otherwise specified.
